# COMPARE CPM-RMI Trial: Intramyocardial Transplantation of Autologous Bone Marrow-Derived CD133+ Cells and MNCs during CABG in Patients with Recent MI: A Phase II/III, Multicenter, Placebo-Controlled, Randomized, Double-Blind Clinical Trial

**DOI:** 10.22074/cellj.2018.6018

**Published:** 2018-05-28

**Authors:** Mohammad Hassan Naseri, Hoda Madani, Seyed Hossein Ahmadi Tafti, Maryam Moshkani Farahani, Davood Kazemi Saleh, Hossein Hosseinnejad, Saeid Hosseini, Sepideh Hekmat, Zargham Hossein Ahmadi, Majid Dehghani, Alireza Saadat, Soura Mardpour, Seyedeh Esmat Hosseini, Maryam Esmaeilzadeh, Hakimeh Sadeghian, Gholamreza Bahoush, Ali Bassi, Ahmad Amin, Roghayeh Fazeli, Yaser Sharafi, Leila Arab, Mansour Movahhed, Saeid Davaran, Narges Ramezanzadeh, Azam Kouhkan, Ali Hezavehei, Mehrnaz Namiri, Fahimeh Kashfi, Ali Akhlaghi, Fattah Sotoodehnejadnematalahi, Ahmad Vosough Dizaji, Hamid Gourabi, Naeema Syedi, Abdolhosein Shahverdi, Hossein Baharvand, Nasser Aghdami

**Affiliations:** 1 Department of Surgery, Baqiyatallah University of Medical Sceinces, Tehran, Iran; 2 Department of Regenerative Medicine, Cell Science Research Center, Royan Institute for Stem Cell Biology and Technology, ACECR, Tehran, Iran; 3 Research Department, Tehran Heart Center, Tehran University of Medical Sciences, Tehran, Iran; 4 Department of Echocardiography, Baqiyatallah University of Medical Sceinces, Tehran, Iran; 5 Department of Cardiology, Baqiyatallah University of Medical Sceinces, Tehran, Iran; 6 Department of Cardiac Surgery, Lavasani Hospital, Social Security Organization, Tehran, Iran; 7 Rajaie Cardiovascular Medical and Research Center, Iran University of Medical Sciences, Tehran, Iran; 8 Department of Nuclear Medicine, Hasheminejad Hospital, Tehran University of Medical Sciences, Tehran, Iran; 9 Transplantation Research Center, NRITLD, Masih Daneshvari Hospital, Shaheed Beheshti University of Medical Science, Darabad, Niavaran, Tehran, Iran; 10 Department of Internal Medicine, Baqiyatallah University of Medical Sceinces, Tehran, Iran; 11 Student Research Committee, School of Nursing and Midwifery , Shahid Beheshti University of Medical Sciences, Tehran, Iran; 12 Echocardiography Research Center, Rajaie Cardiovascular Medical and Research Center , Iran University of Medical Sciences, Tehran, Iran; 13 Department of Pediatrics, Ali Asghar Pediatric Hospital, Tehran University of Medical Sciences, Tehran, Iran; 14 Department of Hematology and Oncology, Firoozgar Hospital, Iran University of Medical Sciences, Tehran, Iran; 15 Department of Heart Failure and Transplantation, Fellowship in Heart Failure and Transplantation, Rajaie Cardiovascular Medical and Research Center, Iran University of Medical Sciences, Tehran, Iran; 16 Department of Internal Medicine, Lavasani Hospital, Social Security Organization, Tehran, Iran; 17 Department of Epidemiology and Reproductive Health, Reproductive Epidemiology Research Center, Royan Institute for Reproductive Biomedicine, ACECR, Tehran, Iran; 18 Department of Reproductive Imaging, Reproductive Biomedicine Research Center, Royan Institute for Reproductive Biomedicine, ACECR, Tehran, Iran; 19 Department of Genetics, Reproductive Biomedicine Research Center, Royan Institute for Reproductive Biomedicine, ACECR, Tehran, Iran; 20 School of Pharmacy and Medical Sciences, Sansom Institute for Health Research, University of South Australia, South Australia, Australia

This article published in Cell J (Yakhteh), Vol 20, No 2, Jul-Sep 2018, on pages 267-277, four affiliations (1, 4, 5, and 10) were changed based on authors request.

